# Melatonin attenuates vascular calcification by inhibiting mitochondria fission via an AMPK/Drp1 signalling pathway

**DOI:** 10.1111/jcmm.15157

**Published:** 2020-05-05

**Authors:** Wei Ren Chen, Yu Jie Zhou, Yuan Sha, Xue Ping Wu, Jia Qi Yang, Fang Liu

**Affiliations:** ^1^ Department of Cardiology Beijing Anzhen Hospital Beijing Institute of Heart Lung and Blood Vessel Disease Beijing Key Laboratory of Precision Medicine of Coronary Atherosclerotic Disease Clinical Center for Coronary Heart Disease Capital Medical University Beijing China; ^2^ Department of Cardiology Nanlou Division Chinese PLA General Hospital National Clinical Research Center for Geriatric Diseases Beijing China

**Keywords:** AMP‐activated protein kinase, Dynamin‐related protein 1, melatonin, mitochondria fission, vascular calcification

## Abstract

Mitochondrial fission plays a role in cardiovascular calcification. Melatonin has previously been shown to protect against cardiovascular disease, so this study sought to explore whether it attenuates vascular calcification by regulating mitochondrial fission via the AMP‐activated protein kinase/dynamin‐related protein 1 (AMPK/Drp1) signalling pathway. The effects of melatonin on vascular calcification were investigated in vascular smooth muscle cells (VSMCs). Calcium deposits were visualized by Alizarin red staining, while calcium content and alkaline phosphatase (ALP) activity were used to evaluate osteogenic differentiation. Western blots were used to measure the expression of runt‐related transcription factor 2 (Runx2), Drp1 and cleaved caspase 3. Melatonin markedly reduced calcium deposition and ALP activity. Runx2 and cleaved caspase 3 were down‐regulated, Drp1 was reduced in response to melatonin, and this was accompanied by decreased apoptosis. Melatonin also reduced levels of mitochondrial superoxide, reversed β‐glycerophosphate (β‐GP)‐induced ΔΨm dissipation and decreased mitochondrial fragmentation. The effects of melatonin in β‐GP‐treated VSMCs were similar to those of mitochondrial division inhibitor 1. Melatonin significantly activated the expression of AMPK and decreased Drp1 expression. Treatment with compound C ablated the observed benefits of melatonin treatment. These findings indicate that melatonin protects VSMCs against calcification by inhibiting mitochondrial fission via the AMPK/Drp1 pathway.

## 1 INTRODUCTION

Vascular calcification (VC) is prevalent in coronary artery disease, and the extent of VC predicts cardiovascular risk.^1^ Causes of calcification in atherosclerosis include dysregulated matrix metabolism, epitaxial mineral deposition, inflammation, oxidative stress and apoptosis.^2^ VC is mainly mediated by vascular smooth muscle cells (VSMCs)^3^ whose transformation from a contractile to osteogenic phenotype promotes the process of VC.^4^ The osteoblastic differentiation of VSMCs is adjusted by the up‐regulation of several osteogenic genes, including runt‐related transcription factor 2 (Runx2), alkaline phosphatase (ALP) and osteocalcin.^5^

Mitochondrial fission plays a role in cardiovascular calcification, and its inhibition was reported to reduce osteogenic differentiation, matrix mineralization and type 1 collagen secretion.^6^ Dynamin‐related protein 1 (Drp1) is a key regulator of mitochondrial fission, and the AMP‐activated protein kinase (AMPK)/Drp1 pathway is associated with mitochondrial fission during cardiovascular disease.^7^, ^8^ Indeed, phosphate‐AMPK protein levels were reported to decrease in VC, while ghrelin improved VC through AMPK activation,^9^ and metformin inhibited β‐glycerophosphate (β‐GP)‐induced impairment of mitochondrial biogenesis via AMPK activation in VC.^10^

Melatonin, the main indoleamine produced by the pineal gland, was recently shown to have anti‐inflammatory, anti‐cancer and antioxidant activities.^11^ Melatonin prevents Drp1‐mediated mitochondrial fission in diabetic hearts^12^ and suppresses Drp1‐mitochondrial fission in human umbilical vein endothelial cells by activation of the AMPK pathway.^13^ The present study aimed to investigate whether melatonin reduces VSMC calcification by inhibiting mitochondrial fission through the AMPK/Drp1 signalling pathway.

## 2 MATERIALS AND METHODS

### 2.1 VSMC isolation, culture and calcification

VSMCs were isolated from the aortas of Sprague Dawley rats (aged 4 weeks) using the explant method described in a previous study.^14^ For calcification, VSMCs were cultured with Dulbecco's Modified Eagle Medium containing 10% foetal bovine serum and 10 mmol/L β‐GP for 14 days.^15^ The medium was changed every 3 days. For drug treatment, melatonin was added before inducing calcification and continued for 14 days. VSMCs were also incubated with mitochondrial division inhibitor 1 (Mdivi‐1, 50 μmol/L) for 14 days.^6^ To evaluate whether the AMPK pathway was involved in the protective effect of melatonin, VSMCs were treated with compound C (1 μmol/L, Sigma‐Aldrich) for 14 days (n = 6 per group in one experiment).^9^ All animal procedures were approved by the Institutional Animal Care and Use Committees at the Capital Medical University.

### 2.2 Measurement of calcium deposition and alkaline phosphatase activity

Cells were fixed with 4% paraformaldehyde for 30 minutes and then stained with 0.1% Alizarin red S (Gefan Biological Technology) for 5 minutes at 37°C. After staining, images were acquired using a Nikon camera. Cells were decalcified with 0.6 mol/L HCl for 24 hours at 37°C, and the calcium contents were determined by the o‐cresolphthalein complexone method (Jiancheng Biological Engineering Institute). ALP activity was measured colorimetrically as the hydrolysis of p‐nitrophenyl phosphate (Beyotime Institute of Biotechnology) and normalized to the total protein content.

### 2.3 MitoSOX red mitochondrial superoxide indicator, mitochondrial membrane potential, mitochondrial permeability transition pore (mPTP) opening and mitochondrial morphology

Cells were washed with cold phosphate‐buffered saline (PBS) and then incubated with MitoSOX Red mitochondrial superoxide indicator (YESEN) at 37°C for 30 minutes. Mitochondrial reactive oxygen species production was analysed using flow cytometry (Sysmex Partec GmbH).

The JC‐1 kit was used to evaluate the mitochondrial membrane potential (Beyotime). Briefly, cells were washed with PBS and incubated with 10 mg/mL JC‐1 at 37°C for 30 minutes. They were then washed a further three times with PBS and observed through fluorescence microscopy. The ratio of JC‐1 aggregate to monomer (red/green) fluorescence was calculated.

The mPTP opening was measured as the rapid dissipation of tetramethylrhodamine ethyl ester fluorescence (TMRE). TMRE fluorescence was alternately excited at wavelengths of 550 nm and 575 nm. The arbitrary mPTP opening time was assessed as the time to the loss of average TMRE fluorescence intensity by half between the initial and residual fluorescence intensity. Cellular ATP levels were measured using a luciferase‐based Enzylight™ ATP assay kit (Beyotime Biotechnology) according to the manufacturer's protocol. ATP contents were normalized to the protein concentration of the resulting supernatant determined with the bicinchoninic acid protein assay kit (Beyotime Biotechnology).

Mitochondrial morphology was assessed using MitoTracker Red images in conjunction with NIH ImageJ software. Mitochondria were observed in at least 100 cells, and their average length was recorded under an inverted microscope (DMI4000; Leica Microsystems).

### 2.4 Western blots

Following experimental treatment, VSMCs were lysed with radioimmunoprecipitation assay lysis buffer containing protease inhibitor (Beyotime Institute of Biotechnology) for 30 minutes and centrifuged at 14 000 *g* for 30 minutes. A bicinchoninic acid protein estimation kit was used to evaluate the protein concentration (Beyotime Institute of Biotechnology). Equal amounts of protein (50 µg) were then loaded into wells of a 10% sodium dodecyl sulphate‐polyacrylamide gel. Proteins were separated by gel electrophoresis and transferred to a polyvinylidene difluoride membrane (Millipore). Membranes were blocked with 5% milk in Tris‐buffered saline containing 0.05% Tween‐20 (TBST) at room temperature for 1 hour followed by overnight incubation at 4°C with the following primary antibodies: anti‐Runx2 (1:1000; Abcam; ab76956), anti‐Drp1 (1:1000, Abcam, ab56788), anti‐pro‐caspase 3 (1:1000, Abcam, ab13847), anti‐cleaved‐caspase 3 (1:1000, Abcam, ab49822), anti‐AMPK (1:1000, Abcam, ab131512), anti‐p‐AMPK (1:1000, Abcam, ab23875) and anti‐β‐actin (1:1000, Abcam, ab8227). After overnight incubation, membranes were washed with TBST and further incubated with an appropriate secondary antibody at room temperature for 1 hour. Membranes were developed with an enhanced chemiluminescence reagent.

### 2.5 Immunofluorescence and TUNEL assays

For immunofluorescence assays, cells were fixed with 4% paraformaldehyde for 30 minutes, followed by permeabilization using 0.5% Triton X‐100 for 10 minutes. Next, cells were blocked with 5% bovine serum albumin for 1 hour and then incubated with a primary antibody against Runx2 (1:200, Cell Signaling Technology), Drp1 (1:200, Cell Signaling Technology), cleaved caspase 3 (1:200, Cell Signaling Technology) or p‐AMPK (1:200, Cell Signaling Technology) overnight at 4°C. The next day, cells were incubated with an appropriate secondary antibody (1:200, Cell Signaling Technology) for 1 hour at 37°C. Images were acquired using a fluorescence microscope (Olympus DX51; Olympus). Apoptosis was detected using a terminal deoxynucleotidyl transferase dUTP nick end labelling (TUNEL) assay (Roche) according to the manufacturer's instructions. The apoptosis index was determined by calculating the percentage of TUNEL‐positive cells to total nucleated cells stained by 4′,6‐diamidino‐2‐phenylindole.

### 2.6 Statistical analysis

Data are described as the mean ± standard deviation (SD) and were analysed by one‐way analysis of variance followed by Tukey's test. The limit of statistical significance between treatment and control groups was *P* < .05.

## 3 RESULTS

### 3.1 Melatonin attenuated β‐GP‐induced VSMC calcification via the suppression of mitochondrial fission

**Figure 1:**
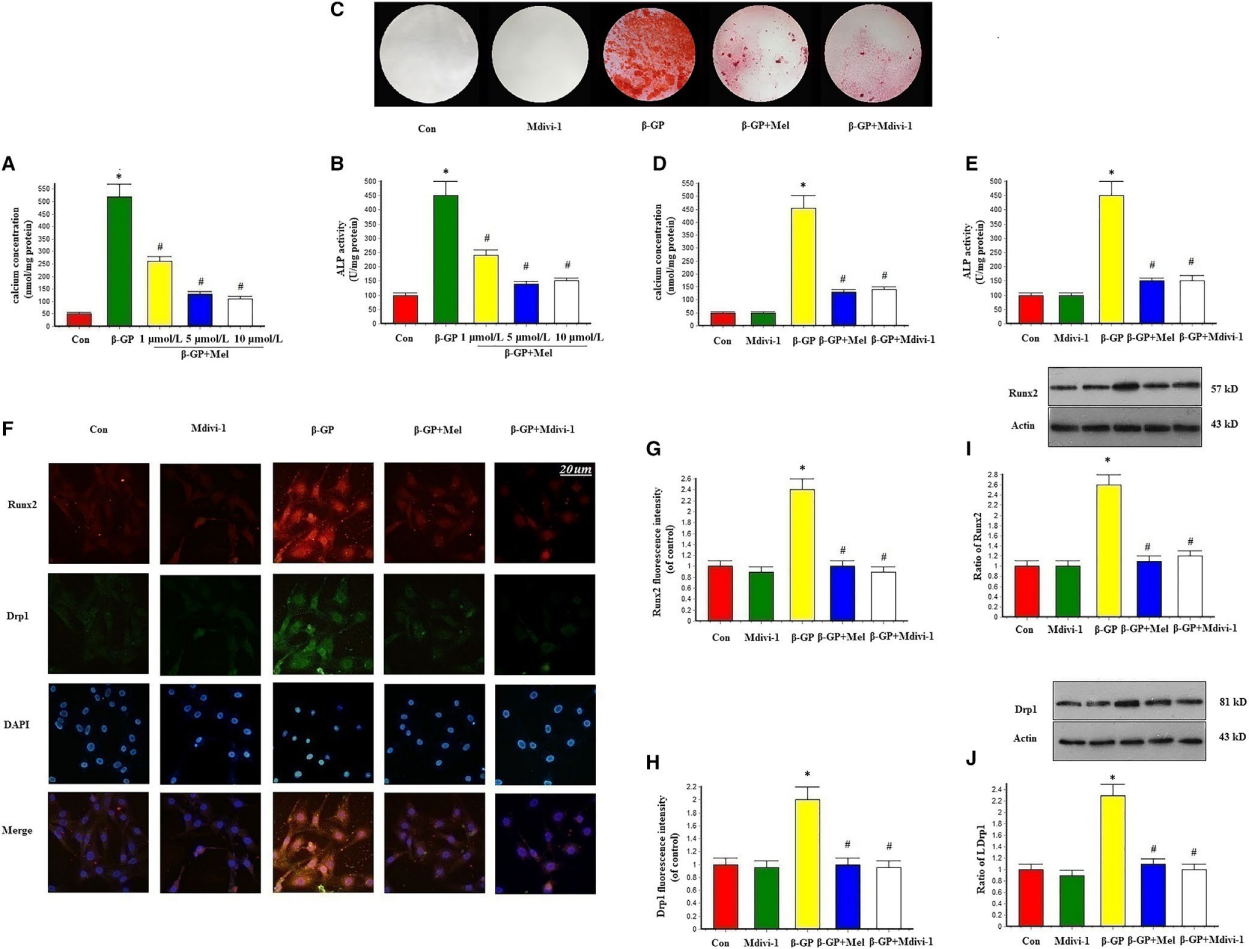
Melatonin reduced β‐GP‐induced calcium deposition via mitochondrial fission inhibition in VSMCs (n = 6/group). VSMCs were cultured with Dulbecco's Modified Eagle Medium containing 10% foetal bovine serum and 10 mmol/L β‐GP for 14 d. A‐B, Result of different concentration of melatonin on calcium content and Alkaline phosphatase (ALP) level. C, Result of melatonin (5 μmol/L) and the mitochondrial division inhibitor 1 (Mdivi‐1, 50 μmol/L) on Alizarin red staining. D, Result of melatonin and Mdivi‐1 on calcium concentration. E, Result of melatonin and Mdivi‐1 on ALP level. F‐H, Result of Immunofluorescence assay (Red signal represents Runx2, and green signal represents Drp1). (I‐J) Results of Runx2 and Drp1 protein expression. *P < .05 vs Con, ^#^ *P* < .05 vs β‐GP

As shown in Figure 1A–B, 5 μmol/L of melatonin significantly reduced calcium content and decreased ALP activity in calcifying VSMCs. Therefore, most experiments were performed using a melatonin concentration of 5 μmol/L. Alizarin Red S staining indicated that β‐GP promoted the calcification of VSMCs, while melatonin significantly inhibited β‐GP‐induced calcification (*P* < .05; Figure 1C–D). Moreover, ALP activity was significantly increased in response to β‐GP, while melatonin significantly reduced ALP activity (Figure 1E). The mitochondrial fission inhibitor Mdivi‐1 also reduced β‐GP‐induced calcification in VSMCs.

An immunofluorescence assay was used to evaluate Runx2 and Drp1 expression in VSMCs. Runx2 protein expression was increased in the β‐GP group, but decreased in the β‐GP and melatonin co‐treatment (β‐GP + melatonin) group. We also found that β‐GP increased Drp1 expression, while melatonin treatment significantly down‐regulated Drp1 expression. Mdivi‐1 treatment reduced Runx2 and Drp1 protein expression, which was comparable to the results in the melatonin group (Figure 1F–H). Western blot results showed that Runx2 and Drp1 expression was similar to that shown in Figure 1F amongst control, β‐GP and β‐GP + melatonin groups (Figure 1I–J).

### 3.2 Melatonin maintained mitochondrial function and structural integrity through mitochondrial fission inhibition

**Figure 2:**
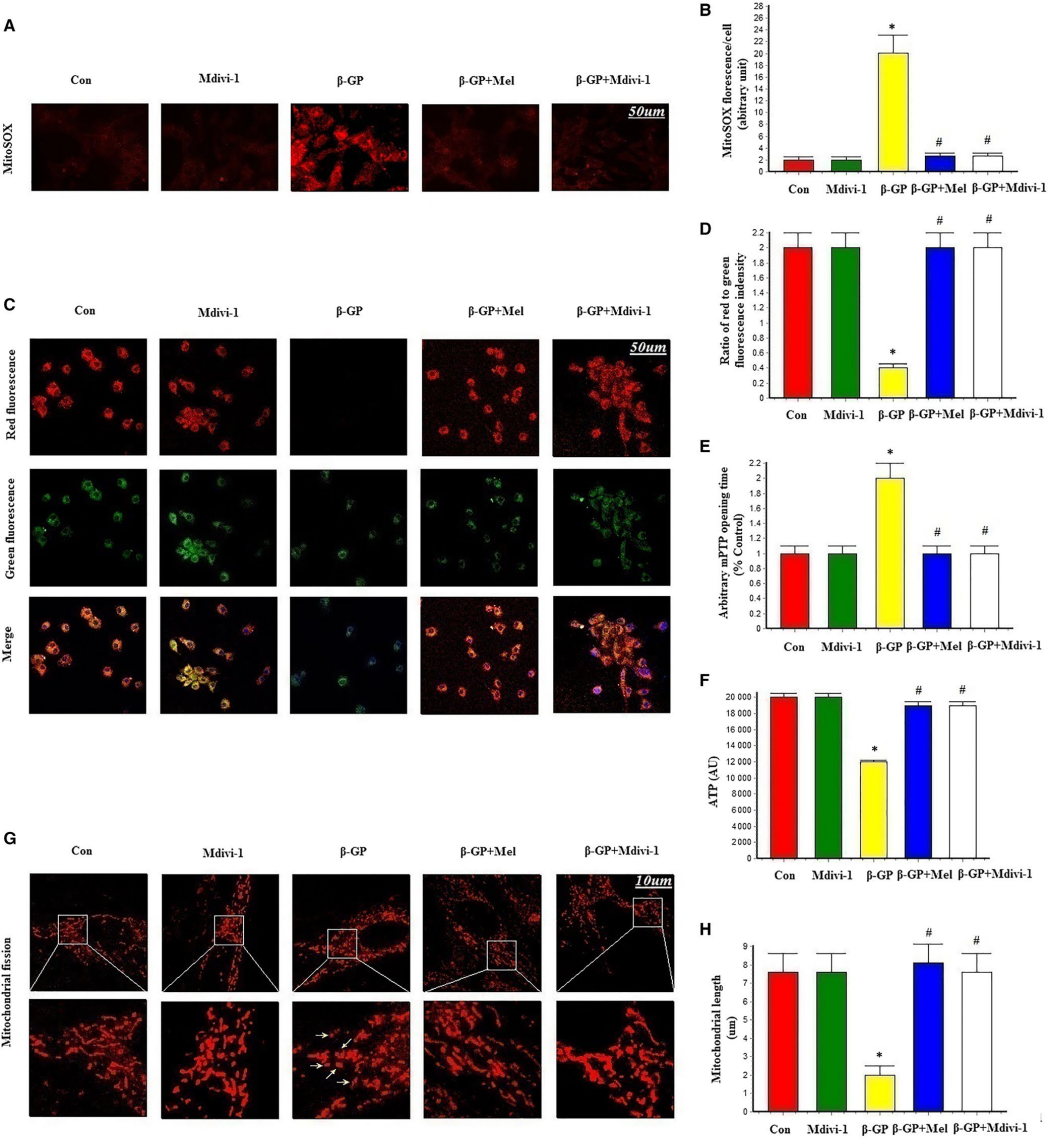
Effects of melatonin on mitochondrial superoxide (MitoSOX), mitochondrial membrane potential, mPTP opening time and mitochondrial fission in VSMCs (n = 6/group). A‐B, MitoSOX for mitochondrial superoxide formation. C‐D, The change of membrane potential (ΔΨm) by JC‐1 staining. E, Result of arbitrary mPTP opening time. F, Result of cellular ATP levels. G‐H, Mitochondrial morphology was observed with the MitoTracker Red. The yellow arrows indicate the fragmented mitochondria. *P < .05 vs Con, ^#^ *P* < .05 vs β‐GP group

To investigate the relationship between melatonin‐mediated vascular protection and oxidative stress, we measured the levels of mitochondrial superoxide and mitochondrial membrane potential (ΔΨm) in VSMCs. β‐GP increased the levels of mitochondrial superoxide, while melatonin and Mdivi‐1 significantly reduced the levels of mitochondrial superoxide (Figure 2A–B). ΔΨm dissipation plays a key role in mitochondrial fission. Here, we found that ΔΨm was dissipated by β‐GP treatment and that melatonin and Mdivi‐1 treatment reversed β‐GP‐induced ΔΨm dissipation (Figure 2C–D). mPTP opening was promoted by treatment with β‐GP; however, this was eliminated by the simultaneous supplementation of melatonin or Mdivi‐1 (Figure 2E). Melatonin and Mdivi‐1 also increased cellular ATP levels after β‐GP treatment (Figure 2F). Mitochondrial fragmentation was significantly increased by β‐GP, but significantly reduced by treatment with melatonin or Mdivi‐1 (*P* < .05; Figure 2G–H). These results suggest that melatonin could maintain mitochondrial function and structural integrity in calcifying VSMCs.

### 3.3 Melatonin protected VSMCs against apoptosis via mitochondrial fission inactivation

**Figure 3:**
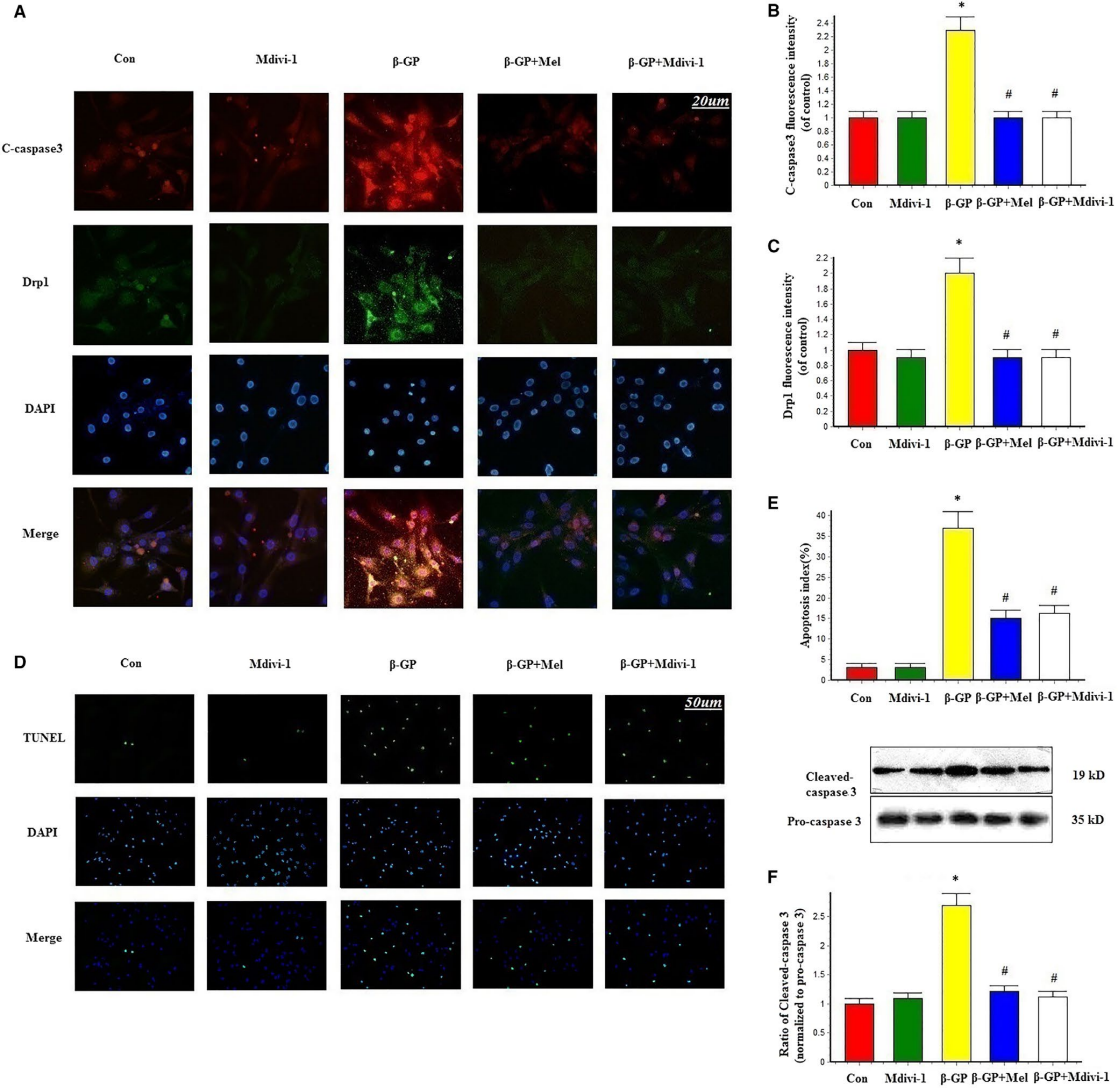
Effects of melatonin on apoptosis in VSMCs (n = 6/group). A‐C, Confocal microscopy of immunofluorescence staining of cleaved caspase 3 (red) and Drp1 (green). D‐E, The apoptosis of VSMC was determined by TUNEL staining. F, Results of cleaved caspase 3 expression. *P < .05 vs Con, ^#^ *P* < .05 vs β‐GP

Immunofluorescence staining results showed that cleaved caspase 3 was increased in the β‐GP group, but decreased in the β‐GP + melatonin group. Mdivi‐1 treatment also significantly reduced cleaved caspase 3 expression compared with the β‐GP group (Figure 3A–C). The TUNEL assay revealed that melatonin and Mdivi‐1 treatment significantly inhibited apoptosis in VSMCs compared with the β‐GP group (Figure 3D–E). Additionally, cleaved caspase 3 protein expression was decreased in the melatonin and Mdivi‐1 groups (Figure 3F). These results indicate that melatonin protected VSMCs against apoptosis via mitochondrial fission inhibition.

### 3.4 Melatonin attenuated β‐GP‐induced VSMC calcification via AMPK signalling

**Figure 4:**
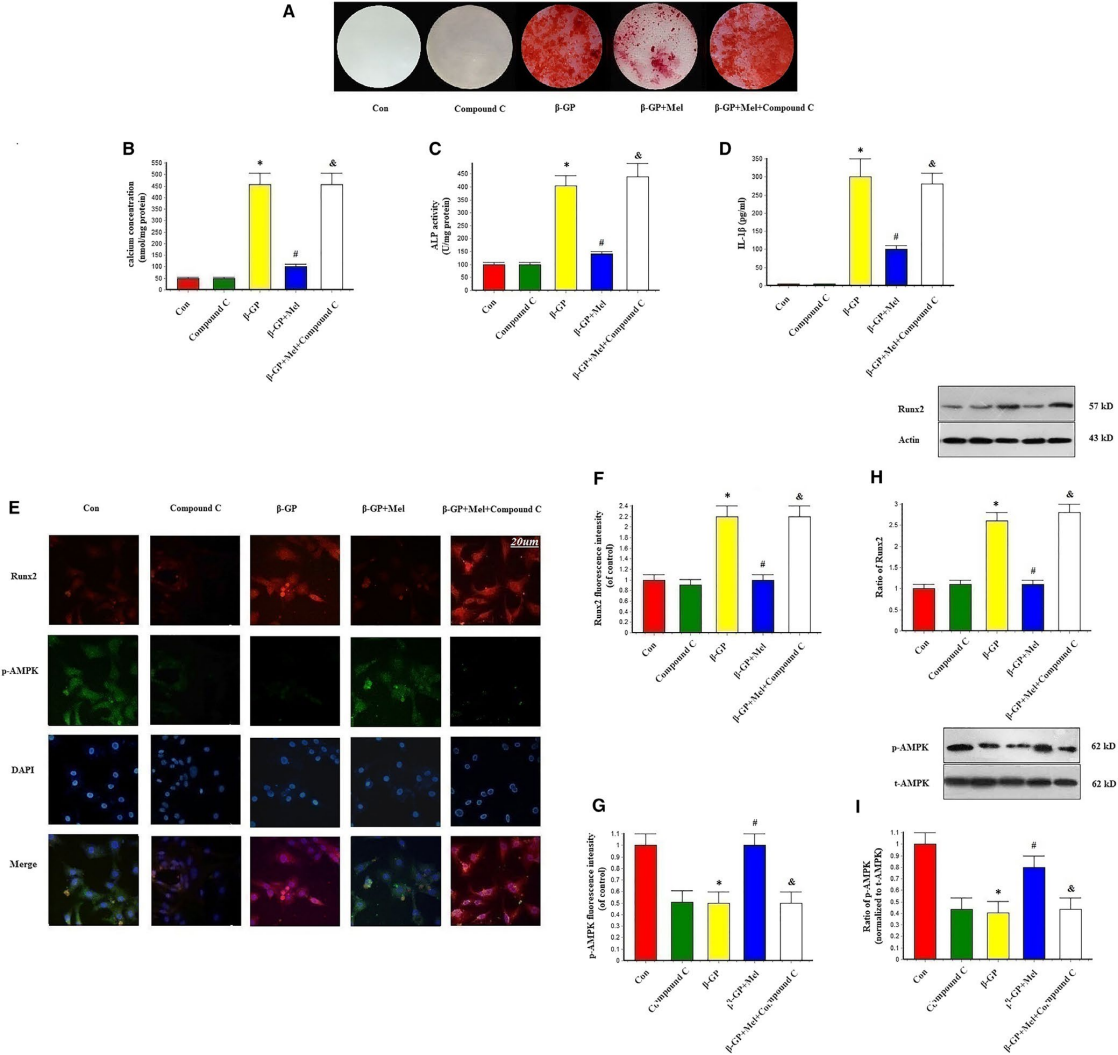
Effects of melatonin and AMPK pathway inhibitor (Compound C, 1 μmol/L) on β‐GP‐induced calcification in VSMCs (n = 6/group). A, Result of Alizarin red staining. B, Result of calcium concentration. C, Result of Alkaline phosphatase (ALP) level. D, Result of interleukin‐1β (IL‐1β) level. E‐G, Results of Immunofluorescence assay (Red signal represents Runx2, and green signal represents p‐AMPK). H‐I, Results of Runx2 and p‐AMPK protein expression. *P < .05 vs Con, ^#^ *P* < .05 vs β‐GP, ^&^ *P* < .05 vs β‐GP + Mel

Melatonin significantly reduced calcium deposition, ALP activity and interleukin‐1β levels in β‐GP‐induced calcified VSMCs. However, the AMPK inhibitor compound C abrogated the protective effects of melatonin on VSMC calcification (Figure 4A–D).

Immunofluorescence revealed that β‐GP promoted Runx2 expression, while melatonin appeared to inhibit this expression; however, this melatonin‐induced inhibition was nullified in the presence of compound C (Figure 4E–G). Additionally, β‐GP increased the expression of Runx2, which was decreased in VSMCs treated with melatonin. However, Runx2 expression levels returned to those observed in the β‐GP group following compound C treatment (Figure 4H–I). These results suggest that melatonin attenuated VSMC calcification through AMPK activation.

### 3.5 Melatonin maintained mitochondrial function and structural integrity via AMPK signalling

**Figure 5:**
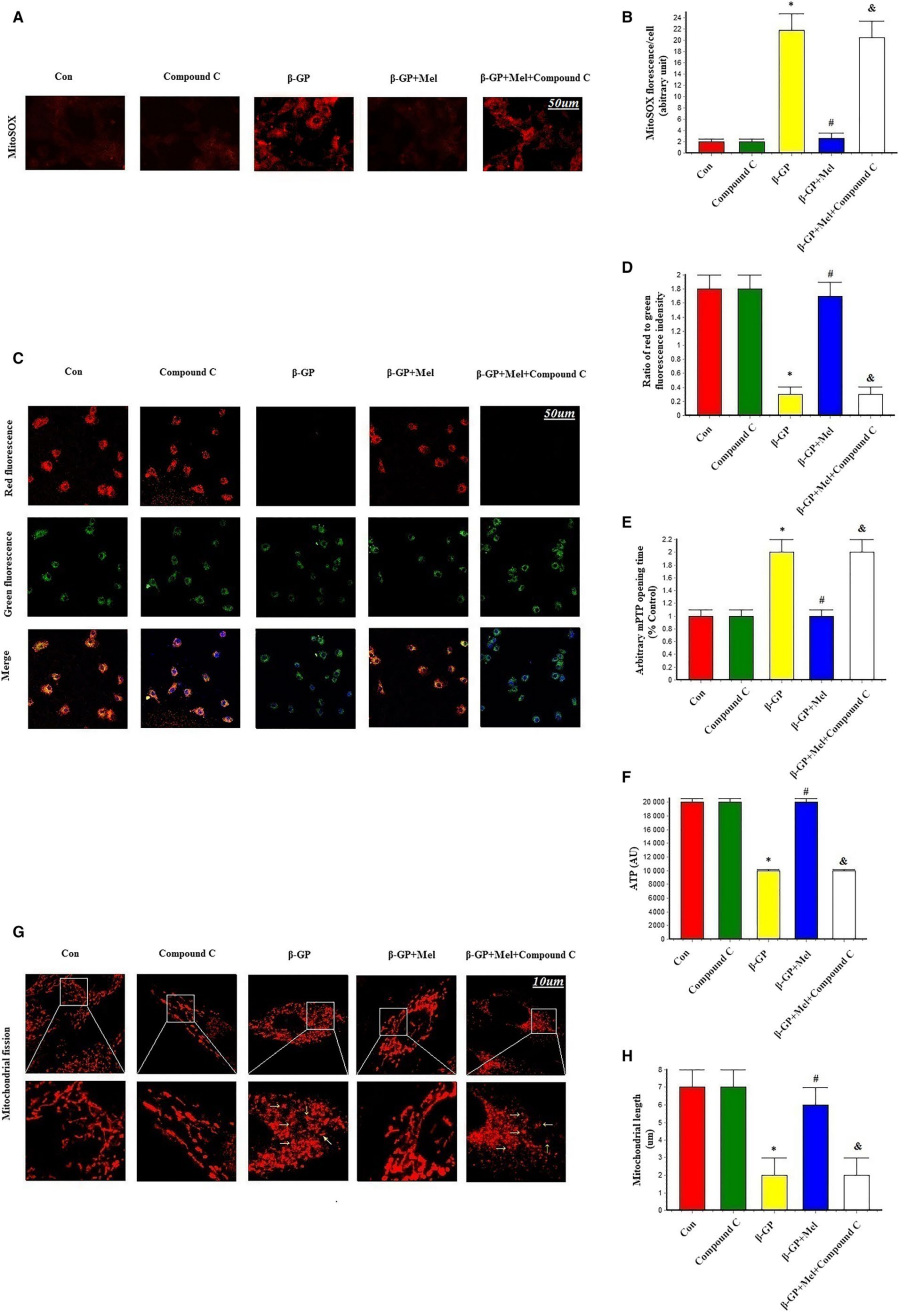
Effects of melatonin and AMPK pathway inhibitor (Compound C, 1 μmol/L) on mitochondrial superoxide (MitoSOX), mitochondrial membrane potential, mPTP opening time and mitochondrial fission in VSMCs (n = 6/group). A‐B, MitoSOX for mitochondrial superoxide formation. C‐D, The change of membrane potential (ΔΨm) by JC‐1 staining. E, Result of arbitrary mPTP opening time. F, Result of cellular ATP levels. G‐H, Mitochondrial morphology was observed with the Mitotracker‐red. The yellow arrows indicate the fragmented mitochondria. *P < .05 vs Con, ^#^ *P* < .05 vs β‐GP group, ^&^ *P* < .05 vs β‐GP + Mel

Melatonin decreased the levels of mitochondrial superoxide, while they were significantly increased by treatment with compound C (*P* < .05; Figure 5A–B). Similarly, β‐GP‐induced ΔΨm dissipation was reversed by melatonin but compound C aggravated the loss of ΔΨm (Figure 5C–D), while the opening of mPTP was inhibited by treatment with melatonin, and this was again negated by compound C (Figure 5E). Compound C also reduced cellular ATP levels in calcifying VSMCs (Figure 5F). Mitochondrial fragmentation was significantly decreased in response to melatonin, but was increased by treatment with compound C (Figure 5G–H). These results suggest that melatonin maintained mitochondrial function and structural integrity through AMPK signalling.

### 3.6 Melatonin protected VSMCs against apoptosis through AMPK signalling

**Figure 6:**
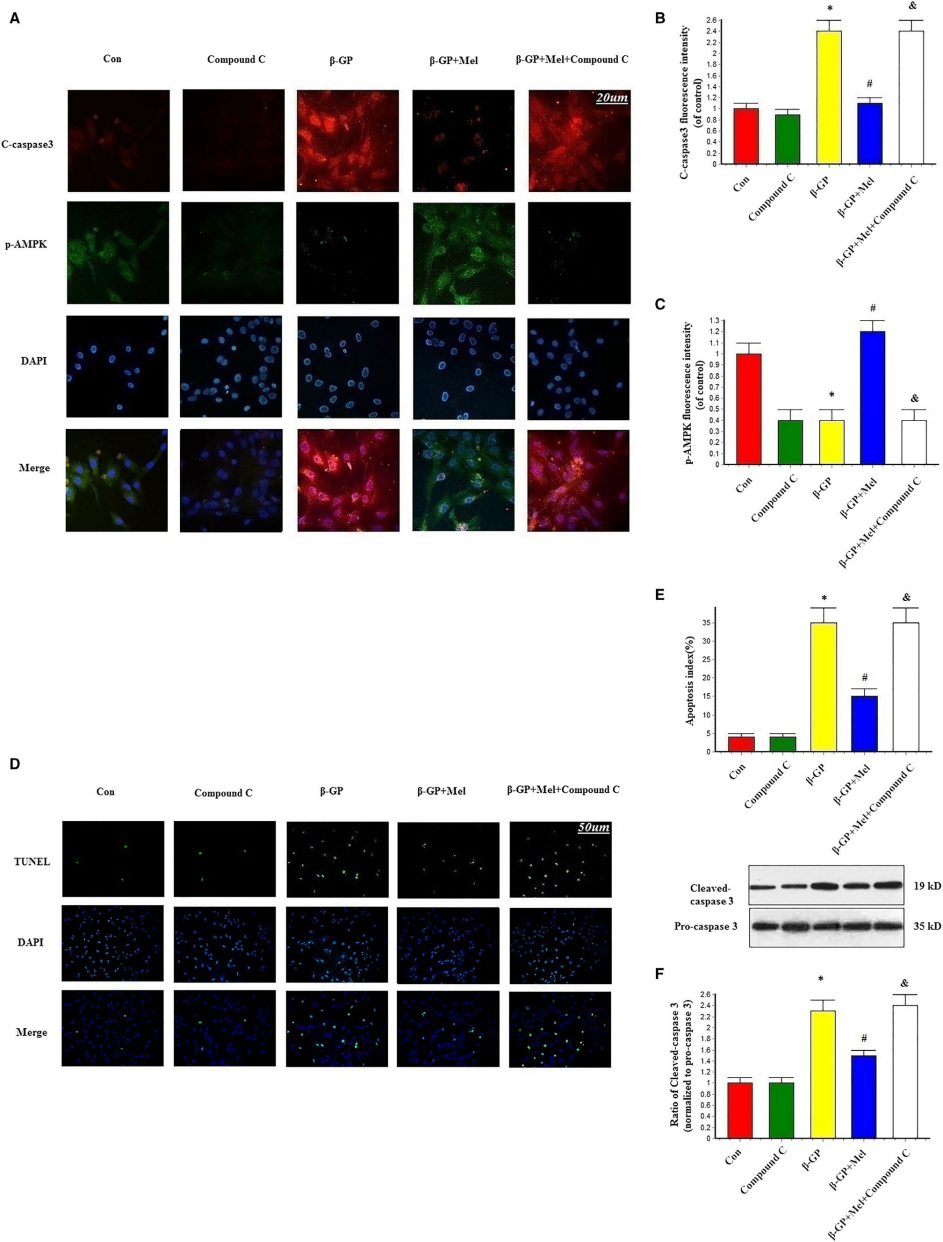
Effects of melatonin and AMPK pathway inhibitor (compound C, 1 μmol/L) on the apoptosis in VSMCs (n = 6/group). A‐C, Confocal microscopy of immunofluorescence staining of cleaved caspase 3 (red) and p‐AMPK (green). D‐E, The apoptosis of VSMC was determined by TUNEL staining. F, Result of cleaved caspase 3 expression. *P < .05 vs Con, ^#^ *P* < .05 vs β‐GP, ^&^ *P* < .05 vs β‐GP + Mel

As demonstrated by the immunofluorescence staining results, cleaved caspase 3 was decreased and p‐AMPK was increased in the melatonin group; however, cleaved caspase 3 was increased and p‐AMPK was decreased in the presence of compound C. This indicated that compound C suppressed AMPK phosphorylation and inhibited the effect of melatonin on apoptosis (Figure 6A–C). This phenomenon was confirmed by Western blot analysis of cleaved caspase 3 expression and TUNEL assays (Figure 6D–F).

### 3.7 Effect of melatonin on AMPK/Drp1 signalling

**Figure 7:**
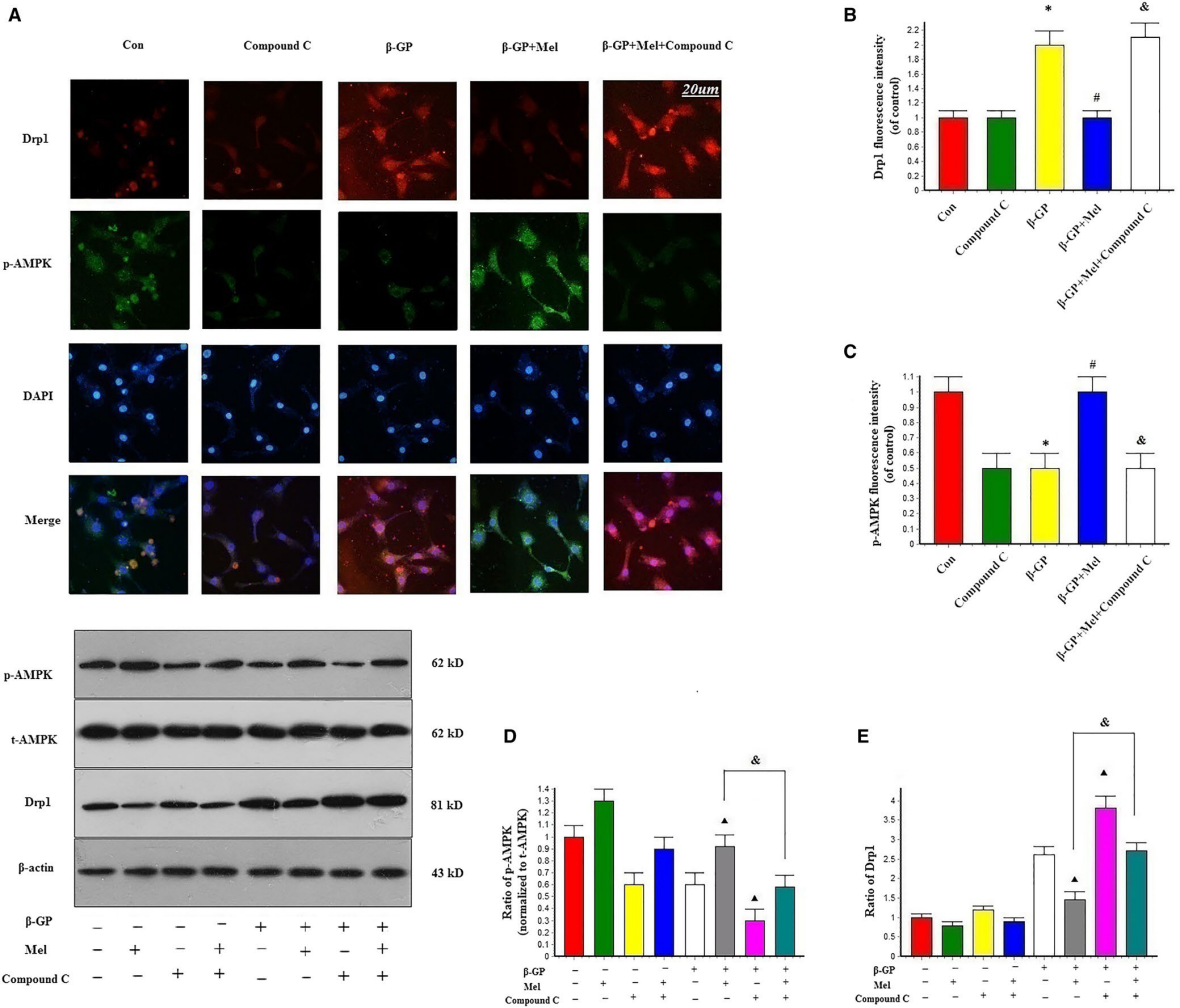
Effects of melatonin on the expression of AMPK and Drp1 in VSMCs (n = 6/group). A‐C, Confocal microscopy of immunofluorescence staining of Drp1 (red) and p‐AMPK (green). *P < .05 vs Con, ^#^ *P* < .05 vs β‐GP, ^&^ *P* < .05 vs β‐GP + Mel. D‐E, The effects of compound C on the expression of p‐AMPK and Drp1. Cells were pre‐treated with or without compound C stimulated with or without melatonin. ^▴^ *P* < .05 vs the values of cells with β‐GP treatment, ^&^ *P* < .05 vs the values of cells treated with melatonin after β‐GP treatment

Immunofluorescence staining also showed that melatonin treatment activated AMPK expression and inhibited mitochondrial fission. Moreover, compound C neutralized the effect of melatonin on mitochondrial fission, as indicated by increased Drp1 and decreased p‐AMPK signals in the compound C‐treated group compared with the melatonin group (Figure 7A–C). Western blotting revealed that melatonin treatment significantly activated the expression of AMPK and decreased Drp1 expression (*P* < .05). Following the application of compound C, a decrease in p‐AMPK was observed and the effects of melatonin on the expression of p‐AMPK and Drp1 following β‐GP‐induced calcification were negated (Figure 7D‐E). These results indicate that melatonin modulated the AMPK/Drp1 signalling pathway.

## 4 DISCUSSION

In the present study, we investigated the effects of melatonin on VSMC calcification and the underlying molecular mechanism. Our results suggest that the observed decrease of VSMC calcification induced by melatonin was mediated, at least in part, by AMPK/Drp1 signalling.

The effect of melatonin on calcification has recently been investigated.^16^, ^17^, ^18^, ^19^ Son et al found that melatonin promoted osteoblastic differentiation and mineralization of preosteoblastic MC3T3‐E1 cells under hypoxic conditions.^16^ However, Kumar and Naidu showed that melatonin significantly antagonized cyclosporine‐induced renal impairment. Microcalcification of the corticomedullary junction subsequent to cyclosporine administration was prevented by melatonin,^17^ while Zhang et al demonstrated that melatonin suppressed oxidative stress‐induced calcification and apoptosis in endplate chondrocytes.^18^ In our previous study, melatonin attenuated β‐GP‐induced calcification of VSMCs, reduced calcium deposition and osteogenic differentiation, and suppressed apoptosis and inflammation.^19^

Mitochondrial fission is involved in many physiological and pathological processes. A study by Rogers et al^6^ showed that Drp1 expression was increased in the osteogenic differentiation of primary human VSMCs, while Drp1 inhibition reduced tissue‐non‐specific alkaline phosphatase activity (a marker of cell differentiation), matrix mineralization and type 1 collagen secretion in VSMC calcification. Drp1 also promoted vascular calcification by regulating osteogenic differentiation. Cui et al found that increased expression and phosphorylation of Drp1 led to the disorganization of cristae, oxidative stress, mitochondria‐mediated apoptosis and subsequent VSMC calcification.^20^ They also found that Mdivi‐1 reversed mitochondrial dysfunction and inhibited VSMC calcification by suppressing oxidative stress and reducing apoptosis.

**Figure 8:**
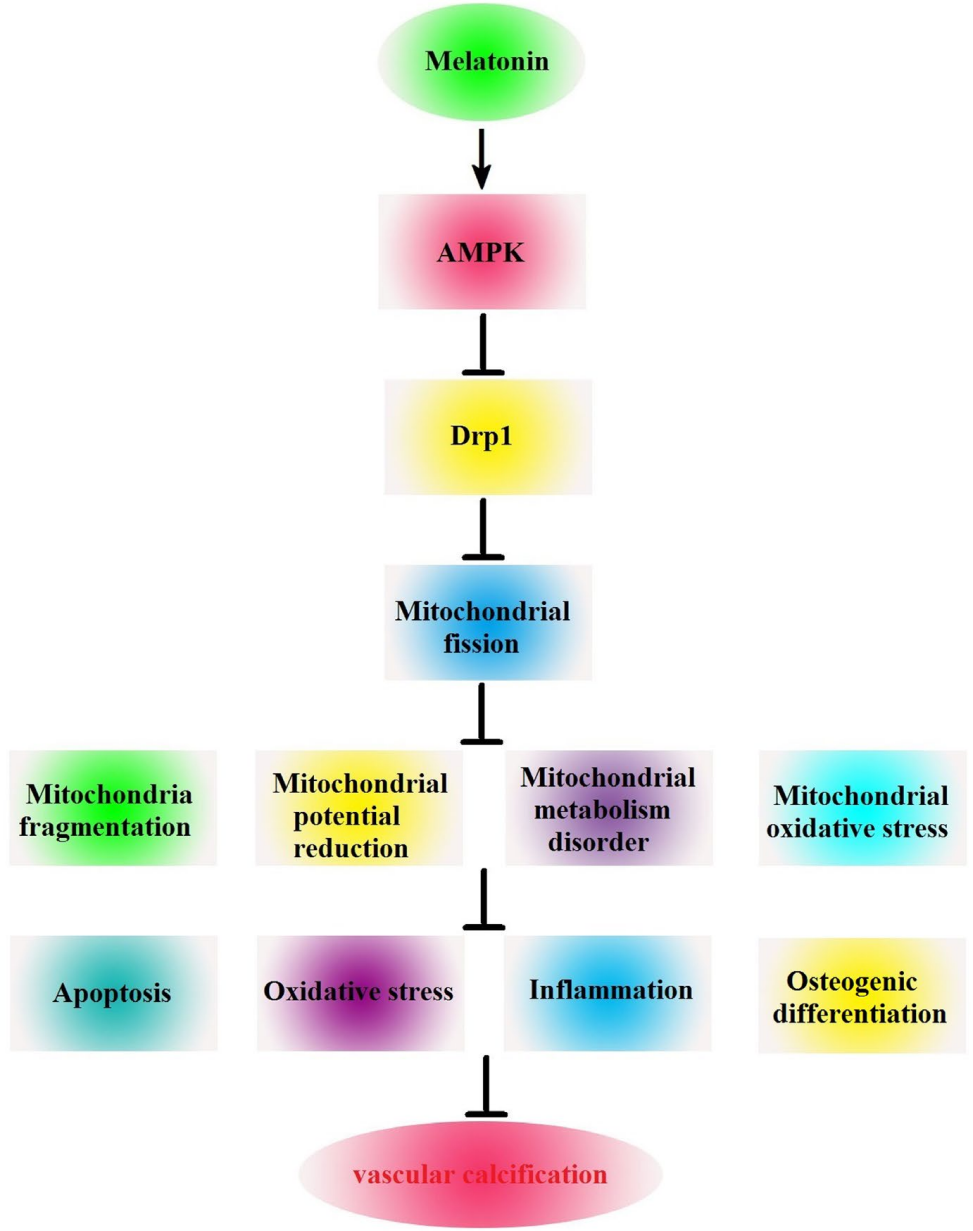
Schematic representation showing that melatonin attenuates VSMC calcification through an AMPK/Drp1 signalling pathway. Melatonin activates AMPK expression, which in turn decreases Drp1 expression and, subsequently, inhibits mitochondrial fission. Suppression of mitochondrial fission reduces apoptosis, oxidative stress, inflammation and osteogenic differentiation. These effects subsequently inhibit VSMC calcification

Mitochondrial fission is associated with AMPK, a key energy sensor that regulates cellular metabolism to maintain energy homeostasis.^21^, ^22^ Kang et al proposed that melatonin induced AMPK phosphorylation and increased mitochondrial fission in rats with carbon tetrachloride‐induced liver fibrosis.^23^ However, Cui et al showed that melatonin treatment reduced the apoptosis of human umbilical vein endothelial cells by inhibiting Drp1‐essential mitochondrial fission through activating the AMPK pathway.^13^ Our experiments demonstrated that melatonin activated AMPK protein expression, inhibited Drp1‐essential mitochondrial fission and reduced VSMC calcification. Furthermore, we found that compound C reduced the effects of melatonin on AMPK/Drp1 and increased VSMC calcification. Taken together, these findings indicate that melatonin activates AMPK expression, which in turn decreases Drp1 expression and, subsequently, inhibits mitochondrial fission. This suppression of mitochondrial fission then reduces apoptosis, reactive oxygen species, Runx2 protein expression and calcium deposition to inhibit VSMC calcification (Figure 8).

The present study has some limitations. First, the findings are only based on in vitro experiments. Second, we did not investigate the effect of knocking down key molecules in the mitochondrial fission process, which would further validate our results. Third, we only observed caspase 3 activation in the current study, so other apoptosis signals should be evaluated in future investigations.

## 5 CONCLUSION

Our study demonstrated that melatonin played an important and protective role in VSMCs by inhibiting calcification via the AMPK/Drp1 system.

## CONFLICT OF INTEREST

The authors declared no potential conflicts of interest with respect to the research, authorship or publication of this article.

## AUTHOR CONTRIBUTIONS

All authors have substantially contributed to the manuscript in terms of conception and design, analysis and interpretation of data, drafting the article, revising it critically for important intellectual content and final approval of the version.

## Data Availability

The data that support the findings of this study are available from the corresponding author upon reasonable request.
